# Optimal control based seizure abatement using patient derived connectivity

**DOI:** 10.3389/fnins.2015.00202

**Published:** 2015-06-03

**Authors:** Peter N. Taylor, Jijju Thomas, Nishant Sinha, Justin Dauwels, Marcus Kaiser, Thomas Thesen, Justin Ruths

**Affiliations:** ^1^Interdisciplinary Computing and Complex BioSystems (ICOS) Research Group, School of Computing Science, Newcastle UniversityNewcastle upon Tyne, UK; ^2^Engineering Systems and Design, Singapore University of Technology and DesignSingapore, Singapore; ^3^School of Electrical and Electronic Engineering, Nanyang Technological UniversitySingapore, Singapore; ^4^Institute of Neuroscience, Newcastle UniversityNewcastle upon Tyne, UK; ^5^Department of Neurology, New York UniversityNew York, NY, USA

**Keywords:** optimal control, numerical methods, epilepsy model, connectome, bistability, spike-wave, stimulation

## Abstract

Epilepsy is a neurological disorder in which patients have recurrent seizures. Seizures occur in conjunction with abnormal electrical brain activity which can be recorded by the electroencephalogram (EEG). Often, this abnormal brain activity consists of high amplitude regular spike-wave oscillations as opposed to low amplitude irregular oscillations in the non-seizure state. Active brain stimulation has been proposed as a method to terminate seizures prematurely, however, a general and widely-applicable approach to optimal stimulation protocols is still lacking. In this study we use a computational model of epileptic spike-wave dynamics to evaluate the effectiveness of a pseudospectral method to simulated seizure abatement. We incorporate brain connectivity derived from magnetic resonance imaging of a subject with idiopathic generalized epilepsy. We find that the pseudospectral method can successfully generate time-varying stimuli that abate simulated seizures, even when including heterogeneous patient specific brain connectivity. The strength of the stimulus required varies in different brain areas. Our results suggest that seizure abatement, modeled as an optimal control problem and solved with the pseudospectral method, offers an attractive approach to treatment for *in vivo* stimulation techniques. Further, if optimal brain stimulation protocols are to be experimentally successful, then the heterogeneity of cortical connectivity should be accounted for in the development of those protocols and thus more spatially localized solutions may be preferable.

## 1. Introduction

Epilepsy is a spectrum of disorders in which patients have seizures due to abnormal neuronal activity (Blumenfeld and Taylor, 2003). Symptomatic manifestations of these events can include a loss of consciousness, tonic-clonic convulsions and myoclonic jerks, amongst others which can severely impact patient quality of life. These transient seizure events often have distinctive electrographic correlates detectable on the electroencephalogram (EEG). One commonly observed electrographic seizure manifestation is the spike wave discharge (SWD). SWDs are high amplitude periodic oscillations with a frequency typically slower then that of normal awake EEG. They are often associated with absence seizures, myoclonic seizures and complex partial seizures (Asconapé and Penry, 1984; Sadleir et al., 2006). Currently the first line of treatment for patients with epilepsy is typically medication, however in over 30% of cases medication alone is insufficient (Keränen et al., 1988).

Brain stimulation has been suggested as an alternative therapeutic treatment for epilepsy (Liang et al., 2010; Berényi et al., 2012; Liang et al., 2012; Saillet et al., 2012). In addition, it has also been suggested that noninvasive stimuli such as an auditory tone (Rajna and Lona, 1989) or through the use of transcranial magnetic stimulation (TMS) (Conte et al., 2007) could be used to interrupt SWD seizures in humans. Unfortunately optimal parameters for stimulation for the abatement of SWD seizures are currently unknown. Attempting to elucidate optimal control parameters in an experimental/clinical setup is difficult due to various ethical, safety and financial reasons.

*In silico* testing of stimulation protocols offers a complementary approach to *in vivo* experimentation. Indeed, several computational models of epileptiform SWD exist at the macroscopic spatial scale which is routinely recorded clinically using EEG. However, many of these models treat the cortex as a spatially continuous homogeneous medium (Robinson et al., 2002; Breakspear et al., 2006; Marten et al., 2009), or disregard spatial interactions altogether (Wang et al., 2012). In contrast, it has been suggested that spatial heterogeneities may be important in seizure genesis or maintenance (Westmijse et al., 2009; Kramer and Cash, 2012; Terry et al., 2012) and should therefore be incorporated into a model (Baier et al., 2012).

Recent years have seen the development of brain imaging protocols using magnetic resonance imaging (MRI) which enable the inference of heterogeneous subject-specific brain connectivity. It is essentially possible to generate a connectivity matrix representing the brain network, with brain areas represented by nodes, and edges/connections inferred using tractography algorithms passing through the white matter. The so-called structural connectome (Sporns et al., 2005), represented as a matrix, can be directly incorporated into a computational model of brain activity. Several previous studies have used this approach to simulate healthy brain function (Honey et al., 2009; Deco et al., 2013; Haimovici et al., 2013; Messé et al., 2014). However, very few have simulated epileptic activity (Taylor et al., 2013b, 2014a; Yan and Li, 2013).

The control of a system with SWD oscillations is highly nontrivial since the system is nonlinear (Taylor et al., 2014b). The goal of seizure abatement through stimulation can be cast as an optimal control problem, which provides a systematic and general approach for designing stimuli. Control theory's traditional analytical techniques, however, do not scale well as the size of the system increases, as is the case in considering a model with spatial heterogeneities. In recent years the pseudospectral method has been applied successfully in a variety of applications as a highly efficient, robust method for the control of large-scale nonlinear systems (Ruths and Li, 2012). In this study we use the pseudospectral method to design time-varying stimuli for SWD seizure abatement *in silico* cast as optimal control problems. The open-loop controls developed by this technique offer distinct advantages in terms of being less invasive and more robust over alternative methods that employ feedback. We test the robustness of our method by applying the approach in different settings. We begin with a relatively simple model which neglects spatial interactions and ultimately build up to large-scale control of a stochastic model using connectivity derived from a patient with clinically diagnosed idiopathic generalized epilepsy. To our knowledge this is the first epilepsy modeling study using patient derived diffusion MRI based connectivity and consequently also the first attempt to control seizures in such a model.

## 2. Materials and methods

### 2.1. Imaging

Cortical connectivity was inferred from a 22 year old female patient clinically diagnosed with idiopathic generalized epilepsy with a history of absence and generalized tonic clonic seizures. The subject gave their written informed consent to participate in this study, which was approved by the Institutional Review Board of NYU Langone School of Medicine. T1 structural MRI and DTI images were acquired using a Siemens Allegra 3T scanner. Diffusion images were collected using 64 directions, with a *b*-factor of 1000 s mm^−2^, one *b*0 image and 2.5 mm isovoxel, TR = 5500 ms, TE = 86 ms. A T1 anatomical image also acquired using the following parameters: TR = 2530 ms, TE = 3.25 ms, FOV = 256 mm at a resolution of 1 × 1 × 1.33 mm.

**Figure 1:**
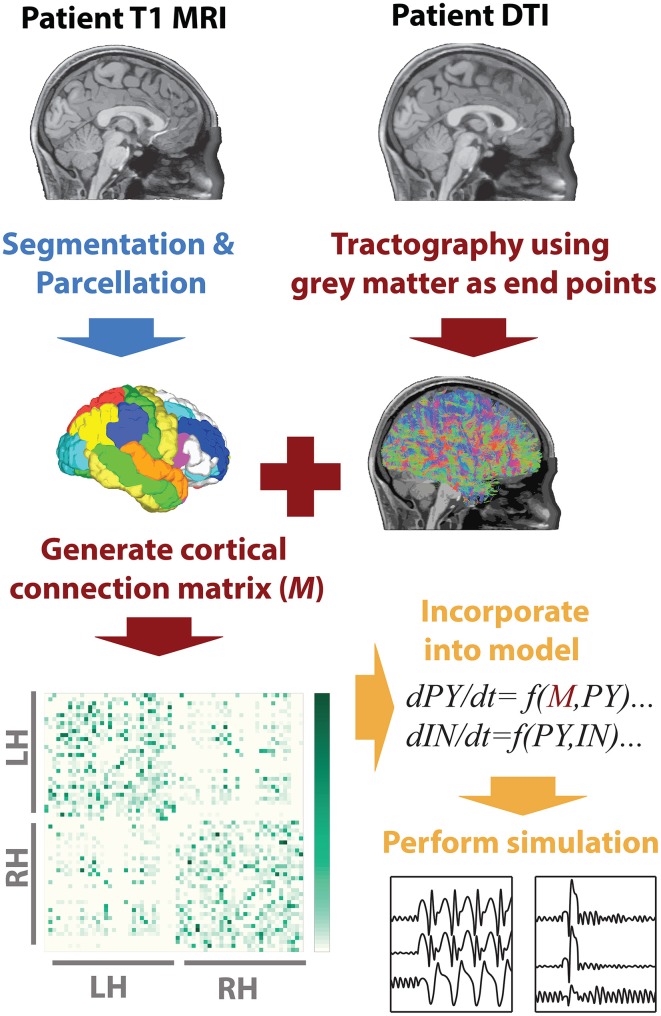
**MRI processing and modeling pipeline**. A patient-specific connectivity matrix is generated using anatomical T1 and diffusion weighted MRI. Segmentation and parcellation are performed using FreeSurfer (blue arrow) to define network nodes and tractography is performed using DSI Studio (red arrows) to determine connections in the network. Custom Matlab code is used to import the connectivity and simulate the model (orange arrows).

To infer the cortico-cortical connectivity of the patient we first, using the T1 image, segmented white matter and gray matter areas, then performed parcellation of the gray matter into 66 regions of interest. These regions of interest correspond to major gyral-based anatomical areas which have been shown to be highly consistent between subjects (Desikan et al., 2006). These gray matter volume ROIs generated using FreeSurfer (http://surfer.nmr.mgh.harvard.edu) were then imported into DSI studio (Yeh et al., 2010) along with the motion corrected diffusion images. Whole brain seeding was then used and tractography was performed. Only tracts with both ends terminating in the gray matter were retained. When a total of 5,000,000 tracts were found tractography was terminated. With the tracts and the ROIs registered to the same space the mean fractional anisotropy along tracts connecting two ROIs was then taken as a connectivity weight. This weighted structural connectivity matrix (*M*) is then used in the model to directly represent cortical connectivity of the patient. Figure 1 summarizes the image processing. A full list of ROI names can be found in Table S1.

### 2.2. Model

#### 2.2.1. Spatially independent

Experimental evidence suggests important roles for both the cortex and thalamus in the genesis and maintenance of epileptic SWD oscillations (Destexhe, 1998; Pinault and O'Brien, 2005). We therefore incorporate knowledge of these anatomical structures into our model using neural field equations based on the Amari framework (Amari, 1977) which has been previously used to model SWD (Taylor and Baier, 2011; Taylor et al., 2014b). The cortical subsystem is composed of excitatory pyramidal (*PY*) and inhibitory interneuron (*IN*) populations. The thalamic subsystem includes variables representing populations of thalmocortical relay cells (*TC*) and neurons located in the reticular nucleus (*RE*). All populations are interconnected in agreement with experimentally known connections (Pinault and O'Brien, 2005) using the connectivity parameters *C*_1…9_. The resulting model equations are therefore:

(1) $$\begin{array}{l} \dot{PY}(t)=\tau_{1}(h_{py}-PY+C_{1}\;f[PY]\\ \;-C_{3}\;f[IN]+C_{9}\;f[TC])+u(t)\\ \dot{IN}(t)=\tau_{2}(h_{in}-IN+C_{2}\;f[PY])+u(t)\\ \dot{TC}(t)=\tau_{3}(h_{tc}-TC-C_{6}\;s[RE]\\ \;+C_{7}\;f[PY])\\ \dot{RE}(t)=\tau_{4}(h_{re}-RE-C_{4}\;s[RE]\\ \;+C_{5}\;s[TC]+C_{8}\;f[PY]) \end{array}$$

where *h_py,in,tc_* are input parameters, τ_1…4_ are timescale parameters and *f*[*x*] is the sigmoid function:

(2) $$f[x]=(1/(1+\epsilon^{-x}))$$

in which *x* = *PY*, *IN*, *TC*, *RE* and ϵ determines the sigmoid steepness. We simplify the thalamic subsystem by using a linear activation term *s*[*x*] = *ax* + *b* instead of the sigmoid function *f*[*x*] since this does not qualitatively impact the dynamics and makes analysis simpler (Taylor et al., 2014b). This follows the connection schematic as shown in Figure S1 based on Pinault and O'Brien (2005).

Deterministic model solutions of Equation (1) are simulated numerically using ode45 in MATLAB. Stochastic model solutions are computed numerically using a fixed step Euler-Maruyama solver in MATLAB with a step size (*h*) of 1/15000 s. Equations for the noise driven system are given in Supplementary Methods Section 1. Stimulations to induce SWD are simulated as a perturbation to the PY and IN variables in state space where the control (stimulus) *u*(t) is applied to the cortical variables only. Parameters are identical to those used in Taylor et al. (2014b).

#### 2.2.2. Spatially extended

Following simulations with only one cortical area, the model can easily be extended to include multiple cortical areas. In our model the cortical areas have local connectivity *within* an area through reciprocal *PY*→*IN* and *IN*⊣*PY* connections in addition to long range excitatory connections only. Long range connections (on the order of several centimeters in length) have been shown experimentally to be predominantly excitatory. We therefore incorporate this into our model using the patient-specific DTI matrix *M* to represent *PY*↔*PY* connections. This approach of incorporating long range connectivity as excitatory is in agreement with previous modeling studies (Babajani-Feremi and Soltanian-Zadeh, 2010) and follows the connectivity schematic and equation in Supplementary Methods Section 2.

### 2.3. Optimal control

Broadly speaking, optimal control is a mathematical framework for systematically selecting the time-varying input needed to drive a dynamical system in a desired way. In general, many choices of input, or stimuli, might achieve a desired objective and without the formalism of optimal control selecting one of these options from a family of potential stimuli is ad-hoc and ill-defined. An optimal control problem couples a cost, or fitness, function to be minimized (or potentially maximized) with a set of constraints. Setting it apart from conventional optimization problems is that this set of constraints includes the differential (or difference) equation that captures the dynamics of the system (Luenberger, 1968). Initial (at the start time, *t* = 0) conditions and often final (at the final time, *t* = *T*) constraints also exist. Path constraints that are imposed over the entire time window *t* ∈ [0, *T*] are also possible. Most critically, the cost function must be selected appropriately to evaluate the candidate options of stimuli and select the correct one.

While the framework of optimal control can capture such a desired objective well, the techniques to solve optimal control problems analytically are limited, especially for large-scale and nonlinear systems. We, therefore, turn to computational methods to solve them. The pseudospectral method is an ideal computational method for this purpose, namely for practitioners in a variety of applied disciplines to use, due to its high level of accuracy and ease of implementation.

The method benefits, like other spectral methods (e.g., Fourier series), from the exponential convergence, as the order of approximation increases, characteristic of orthogonal functions (Fornberg, 1998). In this case we use the Legendre polynomials to approximate the states and control. The method also relies (the “pseudo” part of the name) on a recursive relation between the Lagrange interpolating polynomials and the Legendre polynomials, so that the approximation can be instead approximated by Lagrange polynomials, leading to a double approximation: the unknown states/controls to the Legendre approximation to the Lagrange approximation (Canuto et al., 2006). As the second approximation is an interpolation, the coefficients of the Lagrange approximation are the values of the states and controls themselves at the discretized time points, rather than more abstract coefficients of the Legendre expansion. The latter case (where abstract coefficients are used) is what occurs in a Fourier series approximation of a signal. The coefficients have an interpretation, but the information gleaned is indirect information about the signal itself. These two factors, the *pseudo* and *spectral*, make the method both easy to implement, efficient, and, when combined with standard nonlinear optimization solvers, a powerful and scalable approach for solving optimal control problems.

Ultimately, the pseudospectral method utilizes these approximations to discretize (in time) the continuous optimal control problem into a nonlinear optimization problem. The decision variables of the subsequent optimization problem are the coefficients of the Lagrange interpolating polynomial, which are also the values of the unknown state and control functions at the discretization points. This optimization problem can be solved using any number of commercial or open-source nonlinear solvers. While nonlinear optimization is still a field of much research, the work to-date has produced a number efficient algorithms that scale well on large-scale problems. In order to recover the state and control functions from the discretized solution, we construct the Lagrange approximating polynomial from the optimal decision variables.

A complete presentation of the pseudospectral method and implementation can be found in the supplementary text.

In this work, we use a cost that minimizes the input power (the integrated square of the input). Such a cost function both reduces the invasiveness of the stimuli and also tends to produce inputs that are more interpretable, as they are devoid of non-essential fluctuations in the control shape. We also impose state constraints at the initial and final time to enforce the desired state transfer. Finally, time is discretized into 81 nodes (using a Lagrange approximation of 81 terms), which is dramatically smaller when compared with other methods, such as Runge-Kutta.

## 3. Results

### 3.1. Model dynamics

**Figure 2:**
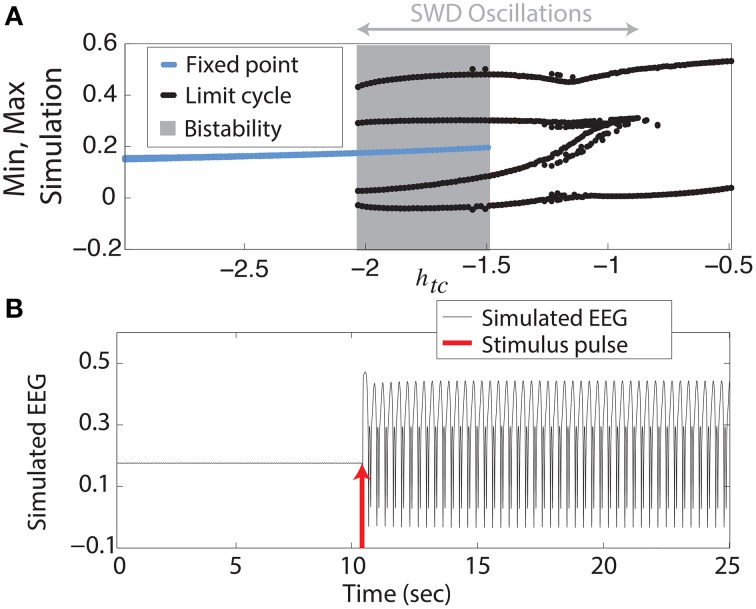
**Bifurcation diagram. (A)** Minima and maxima of time series for different values of *h_tc_*. A fold of cycles bifurcation occurs at the transition between bistability and excitability. **(B)** Time series of the model output.

We begin with the simplest of our scenarios. We investigate the model without noise (i.e., purely deterministic) and independent of any lateral spatial interactions (Equation 1). Figure 2A shows the maxima and minima of the model output for different values of the parameter *h_tc_*. For more negative values shown (*h_tc_* < ≈ −2, left side of figure) there is only one stable solution, all simulations converge to the steady state (stable focus). For less negative values (−2 <≈ *h_tc_* < −1.5, shaded area of figure) a bistable region exists between the stable focus and the SWD oscillations. This arises following a fold of cycles bifurcation at *h_tc_* ≈ −2. Beyond the disappearance of the stable focus (due to a subcritical Hopf bifurcation) at *h_tc_* > −1.5, monostable SWD and slow waves exist (right hand side of figure). In the bistable region a separating manifold (separatrix) exists between the two states in four dimensional state space. This manifold is highly complex in structure (Taylor et al., 2014b).

The stable focus can be considered analogous to resting state background EEG, and the high amplitude oscillatory attractor to be the seizure state (Kalitzin et al., 2010; Taylor et al., 2014b). Transitions between non-seizure and seizure states can occur when a stimulus beyond the separatrix occurs. When this does occur in the bistable region a further stimulus is required to stop the SWD, if a second stimulus is not given the SWD will continue indefinitely. In Figure 2B we show an example time series following such a stimulus. In the region immediately preceding the bifurcation at *h_tc_* ≈ −2 complex excitable transients occur lasting several seconds (Figure S3A). Ultimately the goal of stimulus driven seizure abatement is to minimize the duration of the seizure following detection.

### 3.2. Optimal control of deterministic spike-wave dynamics

The control of SWD implemented here requires a two-step process; seizure detection and seizure control. The seizure is detected when the *PY* and *IN* variables are in the proximity of a point specified on the bistable limit cycle. This could easily be adapted in an experimental setting by using delay embedding to predict state variables (Takens, 1981; Babloyantz and Destexhe, 1986; Taylor et al., 2014b). Since the SWD is fairly regular between cycles and between seizures this “trigger point” can be used, provided that the seizure activity passes close by in state space (e.g., within an error tolerance of ±10%). In theory all points on the SWD limit cycle could be used as trigger points to decrease the time taken to detect and subsequently control the seizure, where each point would correspond to a stimulus with a different profile. This would mean that the stimulus could be applied at any phase in the spike. However, we limit ourselves in this study to a single arbitrarily chosen point and leave optimal seizure *detection* for future study.

**Figure 3:**
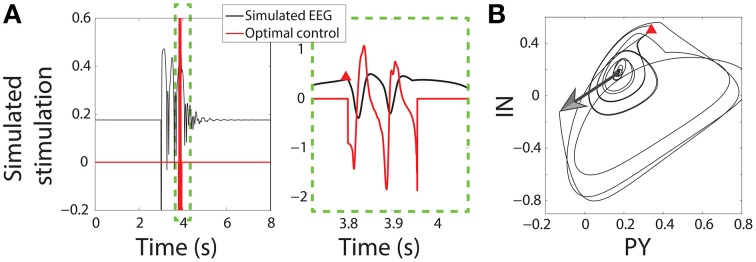
**Control of bistable SWD. (A)** Time series of model and control in the bistable parameter setting (as used in Figure 2). Projection of the PY and IN variables in phase space are shown in **(B)**. Red triangle indicates the trigger point at which the control was applied. The large arrow indicates the stimulus to induce the SWD.

Once the SWD has passed close enough to the trigger point the seizure is detected and the control stimulus is applied starting at that time instant. Figure 3B shows the state space for the PY and IN variables. A stimulus to initiate a seizure is indicated by an arrow, while the red Δ indicates the trigger point. In both the bistable and excitable cases the seizure is abated prematurely by the control (red lines in Figure 3A). An important advantage of the control applied here is that the same control is applied to both the PY and IN variables, while the TC and RE variables are not controlled. This would be desirable in the experimental scenario where a stimulus may activate multiple neuron types with the same waveform morphology and is nonselective. Likewise, stimuli for the TC and RE variables could be developed using the same framework.

Figure 3 shows successful SWD abatement when the model is placed in the bistable setting. Interestingly the same profile can also be used in the excitable transient parameter setting since the flows in state space are similar (Figure S3).

### 3.3. Optimal control of stochastic spike-wave dynamics

**Figure 4:**
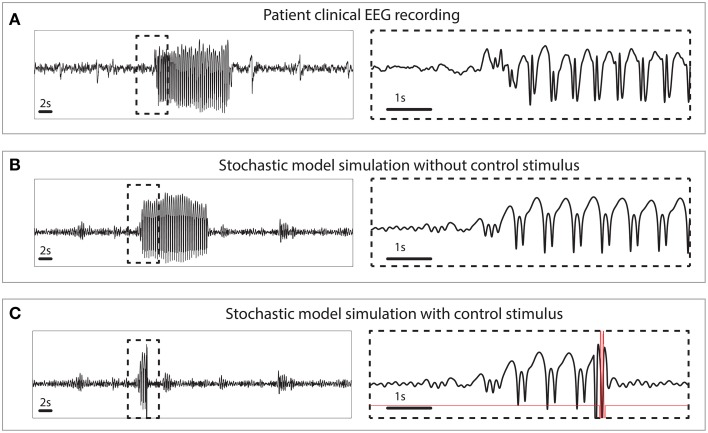
**Clinical and simulated stochastic time series with and without control. (A)** Patient recording from a scalp electrode during a seizure. **(B)** Stochastic model simulation without control. **(C)** Stochastic model simulation with control turned on.

The simulated seizures shown in Figure 3 are artificial in the sense that they are induced by a stimulus at 3 s, indicated by the arrow in state space. In Figure 4 we test the capability of the control stimulus to abate a spontaneously occurring simulated seizure with the inclusion of noise. This has proven extremely challenging in a previous study where noise has been shown to impact the success rate significantly (Taylor et al., 2014b). For comparison, the upper panel of Figure 4 shows a clinical recording of one EEG channel from a patient exhibiting transitions between non-seizure and seizure states. This compares favorably with the stochastic model simulation (Figure 4B). Irregular oscillations around the stable focus driven by noise resemble background activity with an abrupt onset of SWD. Figure 4B shows a simulation without any external control. In Figure 4C, the same control signal in Figure 3 is applied.

The challenge with dealing with stochasticity and the success here with this method underscores the importance of a systematic approach to seizure abatement. Because the optimal control drives the system from near a known trigger point in state space to the background state, the effects of stochasticity are minor. Ad-hoc approaches that work in the deterministic case, may be highly sensitive to the perturbations introduced when noise is added. In previous work (Ruths et al., 2014), we demonstrate how ensemble control can be used to develop stimuli that are robust to variation in the initial state. This situation arises when the noise driven process and the delays in triggering cause the state to shift noticeably before the control can be applied. Because of the consistency of the bistable model, the excitable case requires this extra step of making the stimulus more robust.

### 3.4. Optimal control of heterogeneous spike-wave dynamics

We now apply our method to a case which incorporates patient-derived brain connectivity data. Despite idiopathic generalized epilepsy involving widespread bilateral brain areas, it has been argued that heterogeneity in brain connectivity may contribute to seizure genesis and maintenance (Taylor et al., 2013a). Indeed, it has been suggested that an improved understanding of the heterogeneities involved may lead to more effective treatments for spike-wave seizures (Blumenfeld, 2005). We therefore incorporate patient-specific heterogeneous brain connectivity into our model.

**Figure 5:**
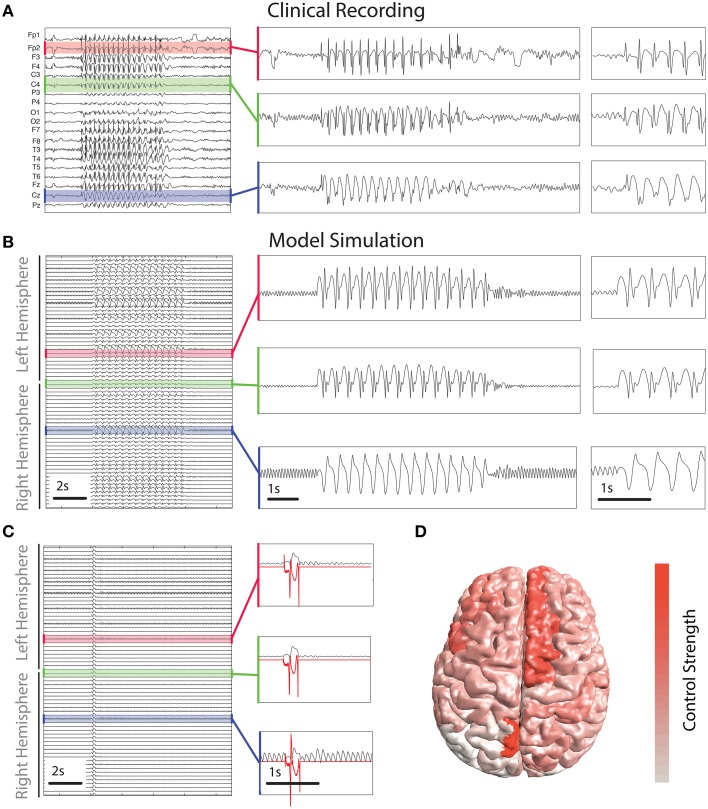
**Control derived using patient-specific connectivity. (A)** Clinical EEG recording of a SWD seizure from 19 scalp electrodes. **(B,C)** show time series of simulated activity without and with the control switched on. Without the control the simulated seizure lasts several seconds. Control is shown in red in **(C)** in three inset panels. **(D)** Spatial distribution of the total strength required to control the seizure. Warmer colors indicate a greater strength is applied in those areas.

For comparison we include a clinical recording of a generalized SWD seizure in Figure 5A. Figure 5B shows a simulation of the model which incorporates the patient based structural connectivity. The model is capable of reproducing various features seen clinically, specifically with respect to spatial variation between recording electrodes. Three simulated channels are zoomed to enable closer examination. They show high, and low amplitude spikes (first two panels) in addition to slow wave oscillations, all of these features are routinely observed clinically (for examples see e.g., Baier et al., 2012 and Figure 5A).

To abate the simulated seizure we apply our optimal control method to all simulated cortical brain areas. Figure 5C shows a time series of a simulated seizure with the control enabled. With the exception of the controls being applied, the model parameters and noise are identical to that shown in Figure 5B. With the control stimuli applied the simulated seizure is terminated almost immediately in all channels. This is despite the spatial heterogeneity in waveform morphology across channels and stimuli. The control signals are shown for three of the simulated brain areas in red in Figure 5C. There are some noticeable differences in morphology and amplitude between the channels. For example, the bottom of the three panels has a much larger positive deflection compared to the other two at the start, while at the end the negative deflection is much weaker. Due to the underlying heterogeneity some brain areas require more total energy to abate (absolute sum of power over time). In essence the total control needs to be stronger for some brain areas than others. Figure 5D shows the strength of stimulus applied for optimal control in different brain areas. Superior frontal areas (more red areas) require more power than occipital areas (more white in color).

## 4. Discussion

In this study we have applied optimal control to a model of epileptiform SWD oscillations incorporating patient-derived connectivity to prematurely abate the simulated seizure. To our knowledge this is the first study to incorporate diffusion MRI based connectivity from a patient into a macroscopic model of epilepsy and also the first attempt at simulating control using a human derived DTI network. We showed that the control can work in different settings (excitable/bistable, stochastic/deterministic) and with different spatial properties (space-independent, heterogeneously spatially-extended).

Previous modeling attempts of seizure control have included several different approaches. One approach is to apply single pulse perturbations in state space beyond the manifold which separates the seizure and non-seizure attractor (Suffczynski et al., 2004; Taylor et al., 2014b). While there is obvious appeal to single pulse stimulation, there are many difficulties with that approach, especially in stochastic systems where repeated success can be troublesome (Taylor et al., 2014b).

A second approach leverages methods from feedback control theory (Kramer et al., 2006; Ching et al., 2012). While feedback control is the hallmark approach to deal with uncertainty, the controls developed through optimal control provide several key advantages. In contrast, much of the work in neuroscience using optimal control has dealt with stylized models that are analytically tractable (Moehlis et al., 2006; Li et al., 2013). Such analytic results provide a unique level of intuition, however, are not scalable to general large scale cases. We differentiate our work in this paper from the existing literature using control theory for neuroscience applications in the following ways. Trigerred stimuli are applied on an “as needed” basis (i.e., only when the SWD reaches a trigger point) in contrast to continuous feedback controllers which are always on. From a patient perspective, this means that neurological function is identical to pretreatment during the times between seizures. In contrast, feedback controllers continue to operate and may as a consequence abate non-pathological neurological activity. While non-feedback methods are often criticized for lack of robustness to noise and parameter uncertainties, recent development in ensemble control allow robust open-loop controllers to be developed and demonstrated in past work with the model used in this paper (Ruths and Li, 2012; Ruths et al., 2014). One limitation of optimal control techniques is that they are highly dependent on the ability of the model to capture the clinically observed EEG. While this is a limitation, models for neurological behavior are consistently improving, and the method for control presented is highly general, so it can be applied to most models developed in the future. The benefit gained from a known model is that the system is transferred reliably between the states of interest (seizure state to background state). The underlying premise of optimal control is that systems have moments in their dynamics when they are most and least susceptible to external influence. The optimization process teases out these susceptible periods and designs the stimulus to take advantage of them. Although feedback control can deliver a stimulus that adapts according to the state, it is typically sub-optimal because it has no such information about susceptibility. Optimal control permits generating stimuli that are minimal by design, so that the stimulus achieves the objective with the lowest, e.g., energy or duration. Finally, the stimuli found through the optimal control process provide intuition on the nature and dynamics of the of the system.

There are several benefits to the control strategy used here. First, only a subset of all variables are controlled, in this case we only control the cortical variables *PY* and *IN*. In the experimental setting this may be desirable because external noninvasive stimuli (e.g., transcranial magnetic stimulation) may not fully penetrate to deep subcortical structures such as the thalamus. In our control of the spatially extended model, the control is optimal in the sense that a cost function is optimized, given the consideration that all cortical variables are available for control. This may be undesirable experimentally as a more spatially localized solution may be sought, effectively reducing the number of locations that require stimulation to abate the seizure. While such an optimal control problem is easy to formulate, solving this mixed-integer problem is challenging on a problem of this size. An important direction of our future work will seek to minimize the number of cortical areas stimulated through a variety of heuristic approaches. A further benefit is that separate controls for each variable do not necessarily need to be developed for each variable. We have demonstrated this throughout, where the same control has been applied to both the *PY* and *IN* populations (see e.g., Figure 4). Additionally, since the control profile is precomputed, the delivery of the control could be applied in real-time when “trigger points” on the SWD cycle are detected.

In this study, the same optimal controls are applied to both *PY* and *IN*, rather than developing different controls for *PY* and for *IN*. In some experimental scenarios, it may be advantageous to differentiate these neuron populations, for example, when using noninvasive stimuli such as TMS if the model does not capture the variables controlled by the stimulus. In other applications this may not be necessary, such as for invasive stimuli like optogenetics—where the specific variables are thought to be well known (Selvaraj et al., 2014). Furthermore, the low dimensionality of SWD oscillations leads us to suggest that only few variables may need to be controlled (Babloyantz and Destexhe, 1986). Nonetheless, the method presented here is adaptable to generating either simultaneous or differentiated control signals for the various neuron populations; this choice is driven based on the manner in which the stimulus interacts with the tissue.

Interestingly the total strength of control required is different in different areas (Figure 5D). Specifically the lingual gyrus, which is important for vision, required high strength bilaterally. We hypothesize this may be due to a hyperexcitability which may exist for photoparoxysmal response, which is common in patients with IGE and absence epilepsy as is the patient studied here. We also find superior frontal areas to require high stimulus strength. Indeed, superior frontal areas are heavily involved in spike-wave seizures with many patients exhibiting frontally abnormal activity in EEG and functional MRI recordings during seizures (Moeller et al., 2008; Bai et al., 2010). While many IGE patients do have high amplitude abnormal frontal activity during seizures, abnormal activity in other areas is often more patient-specific. This stereotypy is present in both the spatial and temporal aspects of the seizures in many patients (Schindler et al., 2011). Indeed, as the seizure patterns exhibit stereotypy, even beyond SWD seizures, so may the optimal control profiles.

One of the assumptions of our study is that the background state coexists with the SWD limit cycle in the state space. This is essentially a different mechanistic assumption to that of a parameter change as in some previous studies (Breakspear et al., 2006). In that case, control of the slowly varying parameter can abate the seizure. In a recent study the modulation of a parameter was implemented as an ultra-slow variable to cause seizure onset and offset (Jirsa et al., 2014). Indeed, our control strategy developed here could easily be applied to such a slow variable as it would be incorporated as a state in an enlarged model.

We have incorporated clinical data into our model in the form of the connectivity, however, a next step is to perform the control stimuli *in vivo*. This could be performed first in animal models of SWD (Meeren et al., 2005), using high strength diffusion MRI to generate high resolution connectivity matrices (Besson et al., 2014). Furthermore, with active perturbation it may be possible to elucidate the directionality of connections (Palma, Hoffmann, Minotti and Kahane, 2013), which would allow for the the application of network control theory (Liu et al., 2011; Ruths and Ruths, 2014).

To summarize, we have demonstrated a nonlinear optimal control technique with application to epilepsy. We have demonstrated its robustness in different settings, ultimately building up to a large scale model of the brain which includes cortical connectivity derived from a patient with idiopathic generalized seizures. We found that due to the heterogeneity in connectivity, there is heterogeneity in the optimal control applied. We therefore suggest this should be considered when applying stimulation to large cortical areas *in vivo* and that spatially localized solutions may consequently be more desirable.

### Conflict of interest statement

The authors declare that the research was conducted in the absence of any commercial or financial relationships that could be construed as a potential conflict of interest.
