# Supplementary material for: Optimal control based seizure abatement using patient derived connectivity
Source: Front Neurosci. 2015 Jun 3;9:202. doi: 10.3389/fnins.2015.00202 (PMC4453481; doi:10.3389/fnins.2015.00202)
Supplement: Supplementary file 1 [file Presentation1.PDF]

Supplementary Material: Optimal control based seizure abatement using patient derived connectivity

Peter Neal Taylor [1,*], Jijju Thomas [2], Nishant Sinha [3], Justin Dauwels [3], Marcus Kaiser [1,4], Thomas Thesen [5] and Justin Ruths [2]

[1] *Interdisciplinary Computing and Complex BioSystems (ICOS) Research Group, School of Computing Science, Newcastle University, Newcastle upon Tyne, UK*
[2] *Engineering Systems and Design, Singapore University of Technology and Design, Singapore*
[3] *School of Electrical and Electronic Engineering, Nanyang Technological University, Singapore*
[4] *Institute of Neuroscience, Newcastle University, Newcastle upon Tyne, UK*
[5] *Department of Neurology, New York University, New York, USA*

Correspondence*:
Corresponding Author
School of Computing Science, Newcastle University, Newcastle upon Tyne, NE1 7RU, UK, peter.taylor@ncl.ac.uk

1 SPATIALLY INDEPENDENT MODEL

In addition to simulating model dynamics deterministically using ode45 in MATLAB we also simulate a stochastic version of the model. The stochastic system receives normally distributed noise input to the TC variable, scaled by the parameter α, representing input from the brain stem. This follows previous models of the thalamocortical loop (**Robinson et al.**, 2002; **Breakspear et al.**, 2006). This described by equation 1:

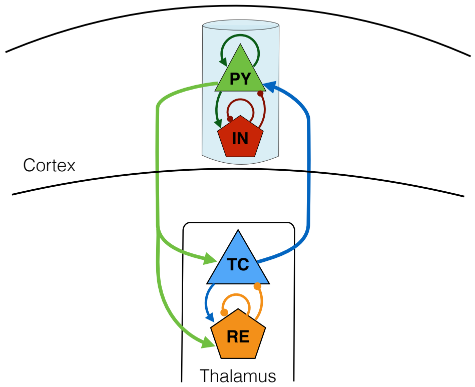

Figure S1. Connectivity scheme of spatially independent model. PY is the cortical pyramidal neural population, IN is the cortical inhibitory neural population, TC is the thalamocortical neural population and RE is the thalamic reticular nucleus neural population. Excitatory (inhibitory) connections for the cortical and thalamic compartments are indicated in green and blue (red and orange) respectively.

$$
\begin{aligned}
dPY(t) &= \tau_1(h_{py} - PY + C_1 f[PY] - C_3 f[IN] + C_9 f[TC] + u_i(t))dt \\
dIN(t) &= \tau_2(h_{in} - IN + C_2 f[PY] + u_i(t))dt \\
dTC(t) &= \tau_3(h_{tc} - TC - C_6 s[RE] + C_7 f[PY])dt \\
dRE(t) &= \tau_4(h_{re} - RE - C_4 s[RE] + C_5 s[TC] + C_8 f[PY])dt
\end{aligned}
\tag{1}
$$

2 SPATIALLY EXTENDED MODEL

The spatially extended two layer neural field model consists of multiple cortical compartments and a thalamic subsystem. Each cortical subsystem is composed of excitatory pyramidal (PY) and inhibitory interneuron (IN) populations. The thalamic subsystem includes variables representing populations of thalamocortical relay cells (TC) and neurons located in the reticular nucleus (RE). Like the spatially independent model, explained in the previous section, the cortico-thalamic connectivities are based on the Amari framework.

Each cortical compartment represents a region of interest. Weighted structural cortico-cortical connectivities are inferred from the diffusion weighted magnetic resonance imaging (M). This heterogeneous connectivity, which is patient specific, is then scaled such that the mean connectivity of all nodes sums to 1. It is then incorporated in the model as the parameter “A”. The stochastic model equations, upon adding a normally distributed noise process W centred around zero and scaled by α, are shown in equation (2).

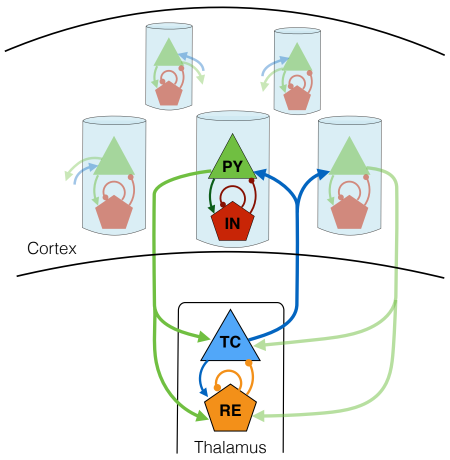

Figure S2. Connectivity scheme of the spatially extended model. Each cortical area illustrates a region of interest. Patient specific long range heterogeneous cortico-cortical anatomical connectivities are inferred using the diffusion MRI (M).

$$\begin{aligned}
dPY_i(t) &= \tau_1(h_{py_i} - PY_i + \sum_{j=1...m} C_1 A_{ji} f[PY_j] - C_3 f[IN_i] + C_9 f[TC] + u_i(t))dt \\
dIN_i(t) &= \tau_2(h_{in} - IN_i + C_2 f[PY_i] + u_i(t))dt \\
dTC(t) &= \tau_3(h_{tc} - TC - C_6 s[RE] + \sum_{j=1...m} C_7 f[PY_j])dt + \alpha_1 dW(t) \\
dRE(t) &= \tau_4(h_{re} - RE - C_4 s[RE] + C_5 s[TC] + \sum_{j=1...m} C_8 f[PY_j])dt
\end{aligned} \tag{2}$$

for $i = 1...m$ with $m = 66$ the number of cortical ROI; $f[X]$ is the sigmoid function,

$$f[X] = (1/(1 + \epsilon^{-X})),$$

and $X = PY_i, IN_i, TC, RE$ with ϵ determines the sigmoid steepness; $\tau_{1 \cdots 4}$ and $h_{py,in,tc,re}$ are the time constants and offset parameters respectively; $C_{1,2,\cdots 9}$ are the connections between PY_i, IN_i, TC, RE populations. This follows the connection schematic shown in Figure S2. Due to the long simulation times of the stochastic model a seizure is induced by perturbing the TC and RE populations.

Heterogeneous values for the parameter h_{py} are used and are offset according to the total incoming connection strength of all other PY populations as defined by equation 3. These heterogeneous values of h_{py} therefore have a mean of -0.35 and standard deviation 0.73 with a range of -2.54 to 1.05 since some brain areas are more connected than others and thus require larger offsets.

$$h_{py_i} = -0.35 - \sum_{j=1...m} A_{ji} - C_1 \tag{3}$$

3 OPTIMAL CONTROL OF EXCITABLE SPIKE-WAVE DYNAMICS

4 OPTIMAL CONTROL

Consider the general optimal control problem

$$\begin{aligned}
\min \quad & \varphi(T, x(T)) + \int_0^T \mathcal{L}(x(t), u(t))\, dt \\
\text{s.t.} \quad & \frac{d}{dt}x(t) = f(x(t), u(t)), \\
& e(x(0), x(T)) = 0, \\
& g(x(t), u(t)) \leq 0,
\end{aligned} \tag{4}$$

where φ and $\mathcal{L}$ are the terminal (evaluated at the terminal time, T) and running cost (depending on the time history of the state and control variables) terms of the general objective function, respectively; f is the system dynamics; e represents endpoint constraints, and g denotes path constraints.

Solving this optimal control problem in the continuous time domain analytically is generally intractable for most complicated systems of interest and we, therefore, turn to computational methods to derive numerical solutions. Our goal is to transform this optimal control problem defined on a function space to a constrained optimization on a vector space.

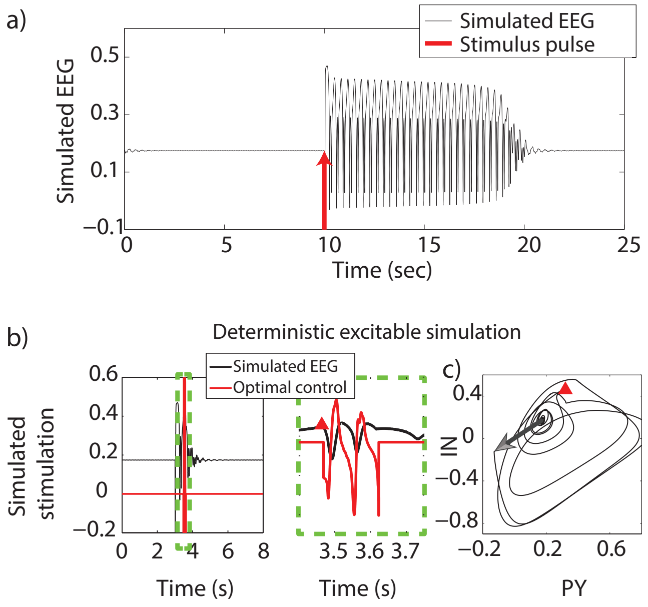

Figure 1. Control of excitable SWD a) Time series of the model following a stimulus to induce SWD. Complex transient SWD persist for several seconds before terminating without further stimuli. b) Time series of model and control in the excitable parameter setting with the control switched on. Projection of the PY and IN variables in phase space are shown in b). Red triangle indicates the trigger point at which the control was applied. The large arrow indicates the stimulus to induce the SWD.

5 PSEUDOSPECTRAL METHOD

The overarching goal of the pseudospectral method is to convert the continuous optimal control problem in (4) into a constrained algebraic minimization problem, which can be solved by efficient nonlinear

Table S1. Parameter values used to produce the figures in the manuscript.

Parameter	Interpretation	Figure 2a	Figure 2b,3	Figure 4	Figure 5
C_1	$PY \to PY$ connectivity strength	1.8	1.8	1.8	1.8
C_2	$PY \to IN$ connectivity strength	4	4	4	4
C_3	$IN \to PY$ connectivity strength	1.5	1.5	1.5	1.5
C_4	$RE \to RE$ connectivity strength	0.2	0.2	0.2	0.2
C_5	$TC \to RE$ connectivity strength	10.5	10.5	10.5	10.5
C_6	$RE \to TC$ connectivity strength	0.6	0.6	0.6	0.6
C_7	$PY \to TC$ connectivity strength	3	3	3	3
C_8	$PY \to RE$ connectivity strength	3	3	3	3
C_9	$TC \to PY$ connectivity strength	1	1	1	1
τ_1	PY timescale	26	26	26	20
τ_2	IN timescale	32.5	32.5	32.5	25
τ_3	TC timescale	2.6	2.6	2.6	2
τ_4	RE timescale	2.6	2.6	2.6	2
h_{py}	Input PY	-0.35	-0.35	-0.35	varies (see eqn. 3)
h_{in}	Input IN	-3.4	-3.4	-3.4	-3.4
h_{tc}	Input TC	varies	-2.0	-2.05	-3.2
h_{re}	Input RE	-5	-5	-5	-5
ϵ	Sigmoid steepness	250000	250000	250000	250000
a	Linear intersection steepness	2.8	2.8	2.8	2.8
b	Linear intersection offset	0.5	0.5	0.5	0.5
α	Standard deviation of noise	0	0	0.022	0.02

numerical optimization solvers. The pseudospectral method was originally developed to solve problems in fluid dynamics and since then has been successfully applied to many areas of science and engineering (**Elnagar et al.**, 1995; **Ross and F. Fahroo**, 2003; **Fahroo and Ross**, 2001).

Pseudospectral discretization methods use expansions of orthogonal polynomials (see Appendix 1.1) to approximate the states of the system and thereby inherit the spectral accuracy characteristic of orthogonal polynomial expansions (the kth coefficient of the expansion decreases faster than any inverse power of k) (**Canuto et al.**, 2006). Through special recursive properties (see Appendix 1.2), derivatives of these orthogonal polynomials can be expressed in terms of the polynomials themselves, making it possible to accurately approximate the differential equation that describes the dynamics with an algebraic relation imposed at a small number of discretization points. An appropriate choice of these discretization points, or nodes, facilitates the approximation of the states as well as ensuring accurate numerical integration through Gaussian quadrature.

We first transform the original problem from the time domain $t \in [0, T]$ to the rescaled domain $t \in [-1, 1]$ on which the orthogonal polynomials are defined. Our choice of the Legendre orthogonal polynomial family suggests we compute the integral term of the cost function using Legendre-Gauss-Lobatto (LGL) quadrature, in which the integral is approximated by a summation of the integrand

Table S2. List of regions of interest obtained after the parcellation of T1 weighted MRI images.

No.	Region of Interest	No.	Region of Interest
1	Left Transverse Temporal	34	Right Transverse Temporal
2	Left Caudal Anterior Cingulate	35	Right Caudal Anterior Cingulate
3	Left Caudal Middle Frontal	36	Right Caudal Middle Frontal
4	Left Cuneus	37	Right Cuneus
5	Left Entorhinal	38	Right Entorhinal
6	Left Frontal Pole	39	Right Frontal Pole
7	Left Fusiform	40	Right Fusiform
8	Left Inferior Parietal	41	Right Inferior Parietal
9	Left Inferior Temporal	42	Right Inferior Temporal
10	Left Isthmus Cingulate	43	Right Isthmus Cingulate
11	Left Lateral Occipital	44	Right Lateral Occipital
12	Left Lateral Orbitofrontal	45	Right Lateral Orbitofrontal
13	Left Lingual	46	Right Lingual
14	Left Medial Orbitofrontal	47	Right Medial Orbitofrontal
15	Left Middle Temporal	48	Right Middle Temporal
16	Left Paracentral	49	Right Paracentral
17	Left Parahippocampal	50	Right Parahippocampal
18	Left Pars Opercularis	51	Right Pars Opercularis
19	Left Pars Orbitalis	52	Right Pars Orbitalis
20	Left Pars Triangularis	53	Right Pars Triangularis
21	Left Pericalcarine	54	Right Pericalcarine
22	Left Postcentral	55	Right Postcentral
23	Left Posterior Cingulate	56	Right Posterior Cingulate
24	Left Precentral	57	Right Precentral
25	Left Precuneus	58	Right Precuneus
26	Left Rostral Anterior Cingulate	59	Right Rostral Anterior Cingulate
27	Left Rostral Middle Frontal	60	Right Rostral Middle Frontal
28	Left Superior Frontal	61	Right Superior Frontal
29	Left Superior Parietal	62	Right Superior Parietal
30	Left Superior Temporal	63	Right Superior Temporal
31	Left Supramarginal	64	Right Supramarginal
32	Left Temporal Pole	65	Right Temporal Pole
33	Left Banks of Superior Temporal Sulcus	66	Right Banks of Superior Temporal Sulcus

evaluated at specific set of nodes,

$$\int_{-1}^{1} f(t)dt \approx \sum_{i=1}^{N} f(t_i)w_i, \qquad w_i = \int_{-1}^{1} \ell_i(t)dt, \tag{5}$$

where N is the order of polynomial approximation, w_i are discrete weights, and $\ell_i(t)$ is the ith Lagrange polynomial, discussed below and in Appendix 1.3 (**Boyd**, 2001). Lobatto in LGL refers to the inclusion of the endpoints as nodes, which is necessary in discretizing optimal control problems in order to enforce initial and terminal conditions. In particular, if the integrand $f \in \mathbb{P}_{2N-1}$ and the nodes $t_i \in \Gamma^{\text{LGL}}$, the integral approximation is exact, where $\mathbb{P}_{2N-1}$ denotes the set of polynomials of degree $2N-1$ or less and where $\Gamma^{\text{LGL}} = \{t_i : L'_N(t)|_{t_i} = 0, i = 1, \ldots N-1\} \bigcup \{-1, 1\}$ are the $N+1$ LGL nodes determined by the derivative of the Nth order Legendre polynomial, $L'_N(t)$, and the interval endpoints (**Canuto et al.**, 2006).

LGL quadrature requires we know the integrand values at the LGL nodes, however, the Nth order Legendre expansions

$$x(t) \approx P_N x(t) = \textstyle\sum_{k=0}^{N} \tilde{x}_k L_k(t), \tag{6}$$

$$u(t) \approx P_N u(t) = \textstyle\sum_{k=0}^{N} \tilde{u}_k L_k(t), \tag{7}$$

do not directly give us a way to discretize the states and controls at these nodes, i.e. the expansions coefficients $\tilde{x}_k$ and $\tilde{u}_k$ have no direct physical meaning. To overcome this, we approximate these Legendre expansions with interpolating polynomials, which, by definition, are equal to the Legendre expansions at the interpolation nodes. Because any interpolating polynomial can be represented by Lagrange polynomials we can represent the state and control as,

$$P_N x(t) \approx I_N x(t) = \textstyle\sum_{k=0}^{N} \bar{x}_k \ell_k(t), \tag{8}$$

$$P_N u(t) \approx I_N u(t) = \textstyle\sum_{k=0}^{N} \bar{u}_k \ell_k(t), \tag{9}$$

where the coefficients $\bar{x}_k$ and $\bar{u}_k$ are the values of the state and control Legendre expansions evaluated at the kth interpolation node, respectively, i.e., $P_N x(t_k) = I_N x(t_k) = \bar{x}_k$ and $P_N u(t_k) = I_N u(t_k) = \bar{u}_k$. The coefficients have this property because the kth Lagrange polynomial is characterized by taking unit value at the kth interpolation node and zero value at all other nodes such that $\ell_k(t_i) = \delta_{ki}$, where δ_{ki} is the Kronecker delta function (**Szegö**, 1959). Using this second approximation we can compute the integrand of the cost function integral at the LGL nodes and $\bar{x}_k$ and $\bar{u}_k$ become the decision variables of the subsequent discrete problem.

Furthermore, the selection of LGL nodes, which are non-uniform on $[-1, 1]$ with quadratic spacing towards the endpoints, as interpolation nodes suppresses the spurious oscillations between nodes that is present when using uniformly spaced nodes, called the Runge phenomena (**Fornberg**, 1998). It can be shown that the LGL interpolation nodes are close to optimal (see Appendix 1.4). The LGL nodes permit us rewrite the Lagrange polynomials in terms of the Legendre polynomials, which is critical to inherit the special derivative and spectral accuracy properties of the orthogonal polynomials despite using Lagrange interpolating polynomials. Given $t_k \in \Gamma^{\text{LGL}}$, we can express the Lagrange polynomial as (see Appendix 1.5) (**Williams**, 2006),

$$\ell_k(t) = \frac{1}{N(N+1)L_N(t_k)} \frac{(t^2-1)L'_N(t)}{t - t_k}. \tag{10}$$

The derivative of (8) at $t_i \in \Gamma^{\text{LGL}}$ is then,

$$\begin{aligned} \frac{d}{dt} I_N x(t_i) &= \sum_{k=0}^{N} \bar{x}_k \dot{\ell}_k(t_i) = \sum_{k=0}^{N} D_{ik} \bar{x}_k \\ &= D_{i0}\bar{x}_0 + D_{i1}\bar{x}_1 + \cdots + D_{iN}\bar{x}_N, \end{aligned} \tag{11}$$

where D is the constant matrix with elements (see Appendix 1.6),

$$D_{ik} = \begin{cases} \frac{L_N(t_i)}{L_N(t_k)} \frac{1}{t_i - t_k} & i \neq k \\ -\frac{N(N+1)}{4} & i = k = 0 \\ \frac{N(N+1)}{4} & i = k = N \\ 0 & \text{otherwise.} \end{cases} \tag{12}$$

We have now effectively discretized all parts of the original optimal control problem. The problem in (4) can now be written as,

$$\begin{aligned} \min \ & \varphi(T, \bar{x}_N) + \frac{T}{2} \sum_{i=0}^{N} \mathcal{L}(\bar{x}_i, \bar{u}_i) w_i \\ \text{s.t.} \ & \sum_{k=0}^{N} D_{ik} \bar{x}_k = \frac{T}{2} f(\bar{x}_i, \bar{u}_i), \\ & e(\bar{x}_0, \bar{x}_N) = 0, \\ & g(\bar{x}_i, \bar{u}_i) \leq 0, \quad \forall\, i \in \{0, 1, \ldots, N\}. \end{aligned} \tag{13}$$

Notice that the second and third lines are equality constraints reflecting the dynamics and endpoint conditions, respectively, and the last line is an inequality constraint reflecting the path constraints.

6 OPTIMAL ENSEMBLE SAMPLING

The ensemble optimal control problem in (14) includes another dimension of continuity in the parameter domain, $s \in \Omega \subset \mathbb{R}^{\text{d}}$, which must be discretized (or sampled) to fit within the constrained minimization method. To reduce the complexity of notation we consider only a single parameter variation, i.e., $d = 1$ and $\Omega = [\underline{s}, \bar{s}]$, however, it is straightforward to extend this to higher dimensions.

$$\begin{aligned} \min \ & \int_{\Omega} \left[\varphi(T, x(T, s)) + \int_0^T \mathcal{L}(x(t, s), u(t))\, dt \right] ds \\ \text{s.t.} \ & \frac{d}{dt} x(t, s) = f(x(t, s), u(t)), \\ & e(x(0, s), x(T, s)) = 0, \\ & g(x(t, s), u(t)) \leq 0, \end{aligned} \tag{14}$$

Consider now the ensemble extension of the interpolation approximation in (8),

$$\begin{aligned} x(t,s) \approx I_{N\times N_s}x(t,s) &= \sum_{k=0}^{N} \bar{x}_k(s)\ell_k(t) \\ &\approx \sum_{k=0}^{N}\left(\sum_{r=0}^{N_s}\bar{x}_{kr}\ell_r(s)\right)\ell_k(t), \end{aligned} \tag{15}$$

and the ensemble extension of the approximate derivative from (11) at $t_i \in \Gamma^{\mathrm{LGL}}$ and $s_j \in \Gamma^{\mathrm{LGL}}_{N_s}$,

$$\begin{aligned} \frac{d}{dt}I_{N\times N_s}x(t_i,s_j) &= \sum_{k=0}^{N} D_{ik}\left(\sum_{r=0}^{N_s}\bar{x}_{kr}\ell_r(s_j)\right) \\ &= \sum_{k=0}^{N} D_{ik}\bar{x}_{kj}, \end{aligned} \tag{16}$$

where $\bar{x}_{kj} = x(t_k, s_j)$. In (15) and (16) we have effectively used a two dimensional interpolating grid at the $N+1$ and N_s+1 LGL nodes in time and the parameter, respectively. Using (15), (16), in conjunction with the LGL quadrature rule, we summarize the ensemble pseudospectral discretization of the ensemble optimal control problem as

$$\begin{aligned} \min\ & \frac{\bar{s}-\underline{s}}{2}\sum_{r=0}^{N_s}\left[\varphi(T,\bar{x}_{Nr}) + \frac{T}{2}\sum_{i=0}^{N}\mathcal{L}(\bar{x}_{ir},\bar{u}_{ir})w_i^N\right]w_r^{N_s} \\ \text{s.t.}\ & \sum_{k=0}^{N} D_{ik}\bar{x}_{kr} = \frac{T}{2}f(\bar{x}_{ir},\bar{u}_i), \\ & e(\bar{x}_{0r},\bar{x}_{Nr}) = 0, \\ & g(\bar{x}_{ir},\bar{u}_{ir}) \leq 0, \quad \forall \begin{array}{c} i \in \{0,1,\ldots,N\} \\ r \in \{0,1,\ldots,N_s\} \end{array}, \end{aligned} \tag{17}$$

where w^N and w^{N_s} are the LGL quadrature weights corresponding to polynomial approximations of order N and N_s respectively. Notice that the summation across the parameter domain in the cost function is multiplied by the interval length. This is because the summation reflects the integral, which is defined on $[-1,1]$.

7 IMPLEMENTATION

Now that the pseudospectral method transforms a continuous optimal control problem to a constrained minimization on a vector space, the problem becomes much more straightforward to solve numerically. We focus here on the AMPL modeling language, however, this can be done in virtually any environment with linear algebra routines and nonlinear optimization capability. AMPL provides a succinct and powerful way of coding these problems which enables them to be input quickly and new problems to be easily adapted from older problems. At the same time AMPL is a gateway to many solvers making it also a powerful optimization choice.

The original problem has unknown functions $x(t) \in \mathbb{R}^n$ (or $x(t, s)$ in the ensemble case) and $u(t) \in \mathbb{R}^m$ and a possibly unknown terminal time T. Approximating the states and controls with polynomials of order N on the nodes Γ_N^{LGL} creates interpolating polynomials with coefficients $\bar{x}_i$ and $\bar{u}_i$

$$\left[\begin{array}{c} x_1(t) \\ \vdots \\ x_n(t) \\ \hline u_1(t) \\ \vdots \\ u_m(t) \end{array}\right] \Rightarrow \left[\begin{array}{cccc} \bar{x}_{10} & \bar{x}_{11} & \cdots & \bar{x}_{1N} \\ \vdots & & & \vdots \\ \bar{x}_{n0} & \bar{x}_{n1} & \cdots & \bar{x}_{nN} \\ \hline \bar{u}_{10} & \bar{u}_{11} & \cdots & \bar{u}_{1N} \\ \vdots & & & \vdots \\ \bar{u}_{m0} & \bar{u}_{m1} & \cdots & \bar{u}_{mN} \end{array}\right]$$

which become the decision variables of the discretized optimization in addition to the terminal time T.

7.1 AMPL SYNTAX

There are only a few key concepts which compose AMPL code. AMPL code is broken into two sections, the model and the data. The model typically describes the form of the problem, but no specific values and leaves these to be specified in the data section. The model is composed of parameters (`param`), variables (`var`), collections on which to define parameters and variables (`set`), a quantity to maximize or minimize (`maximize` or `minimize`), and constraints (`subject to`). These are followed by `data;` and then specifications of values for the parameters and possibly variables for the optimization. There are many excellent AMPL references freely available online. See Appendix 1.7 for examples.

1 APPENDIX

1.1 ORTHOGONAL POLYNOMIALS

Given a non-negative weight function $w(t) \geq 0$, $\int_a^b w(t)dt > 0$, and a weighted inner product $f, g \in L_w^2(a, b)$,

$$\langle f, g \rangle_w = \int_a^b f(t)g(t)w(t)dt,$$

it is possible to create an orthogonal basis, $\{\phi_k\}$, using the Gram-Schmidt process, i.e.,

$$\langle \phi_i, \phi_j \rangle_w \propto \delta_{ij}.$$

Furthermore, orthogonalizing the non-negative powers of t yields a set of orthogonal *polynomials*,

$$(1, t, t^2, \ldots, t^N; w(t)) \Rightarrow \{p_k\}$$

where $p_k \in \mathbb{P}_{\mathbb{N}}$. Legendre polynomials, $\{L_k(t)\}$, are derived with unit weight function, $w(t) = 1$. Therefore, the Gram-Schmidt process for the Legendre polynomials is given by $L_0(t) = 1$ and

$$L_k(t) = t^k - \sum_{i=0}^{k-1} \underbrace{\frac{\langle t^k, L_i(t) \rangle}{\langle L_i(t), L_i(t) \rangle}}_{\text{project } t^k \text{ onto } L_i(t)} L_i(t) = t^k - \sum_{i=0}^{k-1} \frac{\int_{-1}^1 t^k L_i(t)dt}{\int_{-1}^1 L_i^2(t)dt} L_i(t)$$

for $k > 0$. A different orthogonal polynomial family would use a different weight, w, but the process would be similar.

1.2 LEGENDRE POLYNOMIAL PROPERTIES FOR OPTIMAL CONTROL

Recall that the ability of spectral methods to convert a differential equation into an algebraic equation is the feature which makes them powerful tools for problems such as those of optimal control. Legendre polynomials, $L_k(t)$, obey a recursion relation,

$$L_{k+1}(t) = \frac{2k+1}{k+1} t L_k(t) - \frac{k}{k+1} L_{k-1}(t) \tag{18}$$

and also the differential relation,

$$[(1-t^2)L_k'(t)]' + k(k+1)L_k(t) = 0 \tag{19}$$

These two relations illustrate how for function x expanded in terms of L_k, we can express $x'(t) = \dot{x}$ in terms of L_k as well - rather than in terms of L_k'. Hence, $\dot{x} = f(x)$ is now an algebraic equation since both sides can be written as an expansion using L_k as basis functions. Other useful properties of Legendre polynomials are

$$L_k(\pm 1) = (\pm 1)^k \tag{20}$$

$$L_k'(\pm 1) = \frac{(\pm 1)^{k+1} k(k+1)}{2} \tag{21}$$

1.3 LAGRANGE INTERPOLATING POLYNOMIALS

Any interpolating polynomial can be represented by the Lagrange polynomial basis. The kth Lagrange polynomial is characterized by taking unit value at the kth interpolation node and zero at all other nodes, which is effectively a shifted Kronecker delta function, i.e. $\ell_k(t_i) = \delta_{ki}$. The Lagrange polynomials can be written in several ways, but the most transparent is the following fractional product of the interpolation nodes,

$$\ell_k(t) = \frac{(t-t_0)\cdots(t-t_{k-1})(t-t_{k+1})\cdots(t-t_N)}{(t_k-t_0)\cdots(t_k-t_{k-1})(t_k-t_{k+1})\cdots(t_k-t_N)} \tag{22}$$

$$= \prod_{\substack{i=0 \\ i\neq k}}^{N} \frac{(t-t_i)}{(t_k-t_i)} \; . \tag{23}$$

1.4 OPTIMAL INTERPOLATION NODES

The optimality of a specific choice of interpolation nodes can been quantified by

$$||x - I_N x||_\infty \leq (1 + \Lambda_N(\Gamma))||x - p_N^*(x)||_\infty \; , \tag{24}$$

where $p_N^*(x)$ is the best approximating polynomial with respect to the uniform norm and $\Lambda_N(\Gamma)$ is the Lebesgue constant defined by

$$\Lambda_N(\Gamma) = \max_{t\in[-1,1]} \sum_{k=0}^{N} |\ell_k(t)| \; , \tag{25}$$

with $\ell_k(t)$ the kth Lagrange polynomial for the interpolation grid Γ. The Lebesgue constant, then, gives the maximum cumulative excursion from zero of the Lagrange polynomial family along the time axis. Although a closed form for the Lebesgue constant is not in the literature, as $N \to \infty$ the Chebychev-Gauss grid (close to the LGL nodes) yields,

$$\Lambda_N(\Gamma_{\text{CG}}) = \frac{2}{\pi}\log N + \frac{2}{\pi}\Big(\gamma + \log\frac{8}{\pi} - \frac{2}{3}\Big) + O\Big(\frac{1}{\log N}\Big)$$

which in this limit is asymptotic to the Lebesgue constant of the optimal interpolation grid (**Smith**, 2006),

$$\Lambda_N(\Gamma_{\text{CG}}) = \frac{2}{\pi}\log N + \frac{2}{\pi}\Big(\gamma + \log\frac{4}{\pi}\Big) + O\Big(\Big(\frac{\log\log N}{\log N}\Big)^2\Big).$$

1.5 LAGRANGE POLYNOMIAL WRITTEN IN TERMS OF LEGENDRE POLYNOMIAL

Define $w(t) = \prod_{i=0}^{N}(t - t_i)$. Taking the derivative,

$$w'(t) = \sum_{k=0}^{N}\prod_{\substack{i=0\\ i\neq k}}^{N}(t - t_i) \qquad \Rightarrow \qquad w'(t_k) = \prod_{\substack{i=0\\ i\neq k}}^{N}(t_k - t_i) \tag{26}$$

We can now express (22) from Appendix 1.3 as,

$$\ell_k(t) = \frac{w(t)}{(t - t_k)w'(t_k)} \tag{27}$$

Recall that the LG nodes (LGL nodes excluding the endpoints) $\{t_1, \ldots, t_{N-1}\}$ are zeros of $L'_N(t)$, therefore $L'_N(t) = (t - t_1)\ldots(t - t_{N-1})$. We can then write $w(t)$ in terms of the N degree Legendre polynomial.

$$\begin{aligned} w(t) &= (t - t_0)\underbrace{(t - t_1)\ldots(t - t_{N-1})}_{L'_N(t)}(t - t_N) \\ &= (t^2 - 1)L'_N(t) \end{aligned} \tag{28}$$

Combining (28) with the Legendre derivative relation (19) from Appendix 1.2,

$$w'(t_k) = [(t_k^2 - 1)L'_N(t_k)]' = N(N+1)L_N(t_k) \tag{29}$$

Substituting (28) and (29) into (27) we yield an expression for the Lagrange interpolating functions in terms of the Legendre polynomials.

$$\ell_k(t) = \frac{1}{N(N+1)L_N(t_k)}\frac{(t^2 - 1)L'_N(t)}{t - t_k}$$

Once we have the Lagrange polynomials in terms of the Legendre polynomials we can analytically compute the weights for LGL quadrature integration,

$$w_k = \int_{-1}^{1} \ell_k(t)dt = \frac{2}{N(N+1)} \frac{1}{[L_N(t_k)]^2}, \quad i = 0, 1, \ldots, N. \tag{30}$$

1.6 DERIVATIVE MATRIX

$D_{ik} = \dot{\ell}_k(t_i)$ is an $(N+1) \times (N+1)$ matrix. Taking the time derivative of (10), and using the Legendre relation (19) from Appendix 1.2 for the derivative of the numerator yields,

$$\left[\frac{\partial}{\partial t}\ell_k(t)\right]_{t=t_i} = \frac{1}{N(N+1)L_N(t_k)} \left[\frac{N(N+1)L_N(t_i)}{t_i - t_k} - \frac{(t_i^2 - 1)L_N'(t_i)}{(t_i - t_k)^2}\right] \tag{31}$$

For any $i \neq k$, the second term in the brackets is zero, since $t = t_i$ is a zero of $(t^2 - 1)L_N'(t)$. Canceling terms, yields the first component of the derivative matrix in (12). For $i = k$, we utilize l'Hopital's rule for each term (we use the rule twice for the second term).

$$\lim_{t \to t_k} \frac{N(N+1)L_N(t)}{(t - t_k)} = \lim_{t \to t_k} \frac{N(N+1)L_N'(t)}{1} = N(N+1)L_N'(t_k) \tag{32}$$

$$\lim_{t \to t_k} \frac{(t^2 - 1)L_N'(t)}{(t - t_k)^2} = \lim_{t \to t_k} \frac{N(N+1)L_N(t)}{2(t - t_k)} = \lim_{t \to t_k} \frac{N(N+1)L_N'(t)}{2} = \frac{N(N+1)L_N'(t_k)}{2} \tag{33}$$

For $i = k \neq 0, N$, $L_N'(t_k) = 0$ which indicates that $\dot{\ell}_k(t_k) = 0$ if $k \neq 0, N$. Substituting the values of $L_N(\pm 1)$ and $L_N'(\pm 1)$ given by equations (20-21) in Appendix 1.2 and combining these two terms,

$$\frac{\partial \ell_k(t_k)}{\partial t} = (\pm 1)\frac{N(N+1)}{4} \qquad k \in \{0, N\} \tag{34}$$

Therefore, the elements of D are as given in (12),

$$D_{ik} = \begin{cases} \frac{L_N(t_i)}{L_N(t_k)} \frac{1}{t_i - t_k} & i \neq k \\ -\frac{N(N+1)}{4} & i = k = 0 \\ \frac{N(N+1)}{4} & i = k = N \\ 0 & \text{otherwise.} \end{cases}$$

The matrix D is the first differentiation matrix. Optimal control requires only the first derivative, as the differential equation is of order 1. Similar expressions can be computed for second, third, etc. differentiation matrices.

1.7 AMPL EXAMPLES

1.7.1 Single Spin Bloch Optimization The following example comes from quantum control.

Problem Definition: $\max\ x(T), T = \pi/2$, subject to $u(t)^2 + v(t)^2 \leq 1, t \in [0, T]$ and

$$\frac{d}{dt}\begin{bmatrix} x \\ y \\ z \end{bmatrix} = \begin{bmatrix} 0 & 0 & u \\ 0 & 0 & -v \\ -u & v & 0 \end{bmatrix}\begin{bmatrix} x \\ y \\ z \end{bmatrix}, \qquad \begin{bmatrix} x(0) \\ y(0) \\ z(0) \end{bmatrix} = \begin{bmatrix} 0 \\ 0 \\ 1 \end{bmatrix}. \tag{35}$$

```
param N > 0 integer;
param A > 0;
param T > 0;

param x0;
param y0;
param z0;

set nodes := 1..(N+1);

param D {nodes,nodes};

var x {nodes} >= -1, <= 1;
var y {nodes} >= -1, <= 1;
var z {nodes} >= -1, <= 1;

var u {nodes} >=-A, <=A;
var v {nodes} >=-A, <=A;

maximize cost: x[N+1];

subject to dynamics_x {t in nodes}:
    u[t]*z[t] = (2/T)*(sum{k in nodes} D[t,k]*x[k]);

subject to dynamics_y {t in nodes}:
    -v[t]*z[t] = (2/T)*(sum{k in nodes} D[t,k]*y[k]);

subject to dynamics_z {t in nodes}:
    -u[t]*x[t] + v[t]*y[t] = (2/T)*(sum{k in nodes} D[t,k]*z[k]);

subject to initialConditions_x: x[1] = x0;
subject to initialConditions_y: y[1] = y0;
subject to initialConditions_z: z[1] = z0;

subject to amplitudeBound {t in nodes}: u[t]^2+v[t]^2 <= A^2;

data;
param N := 10;
param A := 1;
param T := pi/2;

param x0 := 0;
param y0 := 0;
```

```
param z0 := 1;

param D :
    1    2     3     4     5     6     7     8     9     10     11 :=
    1    -27.50   37.20  -14.88   8.49  -5.64   4.06  -3.06   ...
    2     -6.17   0.00     8.73  -4.07   2.53  -1.77   1.31   ...
    3      1.44  -5.11     0.00   5.25  -2.53   1.61  -1.14   ...
    4      ...
    5      ...
    6      ...
    7      ...
    8      ...
    9      ...
    10     ...
    11     0.50  -1.26    1.79   -2.35   3.06  -4.06   5.64  ...
;
```

The D matrix is too large to present in this format, so the ellipses must be replaced with the rest of the matrix values.

1.7.2 Broadband Spin Bloch Optimization The following is an ensemble extension of the previous example.

Problem Definition: $\max \int_\Omega x(T,\omega)d\omega$, $0 \le T \le 2\pi$, $\Omega = [-1,1]$ subject to $u(t)^2 + v(t)^2 \le 1, t \in [0,T]$,

$$\frac{d}{dt}\begin{bmatrix} x(t,\omega) \\ y(t,\omega) \\ z(t,\omega) \end{bmatrix} = \begin{bmatrix} 0 & -\omega & u \\ \omega & 0 & -v \\ -u & v & 0 \end{bmatrix}\begin{bmatrix} x(t,\omega) \\ y(t,\omega) \\ z(t,\omega) \end{bmatrix}, \qquad \begin{bmatrix} x(0,\omega) \\ y(0,\omega) \\ z(0,\omega) \end{bmatrix} = \begin{bmatrix} 0 \\ 0 \\ 1 \end{bmatrix}. \tag{36}$$

```
param N > 0 integer;
param Nw > 0 integer;
param A > 0;
param B > 0;
param Tmax > 0;

param x0;
param y0;
param z0;

set states := 1..3;
set nodes := 1..(N+1);
set dispersion := 1..(Nw+1)

param D {nodes,nodes};
param w {dispersion};
param wwts {dispersion};

var T >= 0 <= Tmax;
var M {states,dispersion,nodes} >= -1, <= 1;

var u {nodes} >=-A, <=A;
var v {nodes} >=-A, <=A;
```

```

maximize cost: ((2*B)/2)*(sum{i in dispersion} M[1,i,N+1]*wwts[i]);

subject to dynamics_x {i in dispersion, t in nodes}:
 -w[i]*M[2,i,t] + u[t]*M[3,i,t] = (2/T)*(sum{k in nodes} D[t,k]*M[1,i,k]);

subject to dynamics_y {i in dispersion, t in nodes}:
 w[i]*M[1,i,t] - v[t]*M[3,i,t] = (2/T)*(sum{k in nodes} D[t,k]*M[2,i,k]);

subject to dynamics_z {i in dispersion, t in nodes}:
 -u[t]*M[1,i,t] + v[t]*M[2,i,t] = (2/T)*(sum{k in nodes} D[t,k]*M[3,i,k]);

subject to initialConditions_x {i in dispersion}:
    M[1,i,1] = x0;
subject to initialConditions_y {i in dispersion}:
    M[2,i,1] = y0;
subject to initialConditions_z {i in dispersion}:
    M[3,i,1] = z0;

subject to amplitudeBound {t in nodes}: u[t]^2+v[t]^2 <= A^2;

data;
param N := 10;
param Nw := 4;
param A := 1;
param B := 1;
param Tmax := 2*pi;

param x0 := 0;
param y0 := 0;
param z0 := 1;

param w :=
  1   -1
  2   -0.5
  3   0
  4   0.5
  5   1
;

param wwts :=
  1   0.1
  2   0.54
  3   0.71
  4   0.54
  5   0.1
;

param D :
    ...
;
```

REFERENCES

Boyd, J. P. (2001), Chebyshev and Fourier spectral methods (Courier Corporation)

Breakspear, M., Roberts, J., Terry, J., Rodrigues, S., Mahant, N., and Robinson, P. (2006), A unifying explanation of primary generalized seizures through nonlinear brain modeling and bifurcation analysis, *Cerebral Cortex*, 16, 9, 1296

Canuto, C., Hussaini, M. Y., Quarteroni, A., and Zang, T. A. (2006), Spectral Methods (Springer)

Elnagar, G., Kazemi, M. A., and Razzaghi, M. (1995), The pseudospectral legendre method for discretizing optimal control problems, *IEEE Transactions on Automatic Control*, 40, 1973–1976

Fahroo, F. and Ross, I. M. (2001), Costate estimation by a legendre pseudospectral method, *Journal of Guidance, Control, and Dynamics*, 24, 2, 270–277

Fornberg, B. (1998), A practical guide to pseudospectral methods, volume 1 (Cambridge university press)

Robinson, P. A., Rennie, C. J., and Rowe, D. L. (2002), Dynamics of large-scale brain activity in normal arousal states and epileptic seizures, *Physical Review E*, 65, 4, 041924

Ross, I. and F. Fahroo, F. (2003), New Trends in Nonlinear Dynamics and Control (Springer, Berlin)

Smith, S. J. (2006), Lebesgue constants in polynomial interpolation, in Annales Mathematicae et Informaticae, volume 33 (Eszterházy Károly College, Institute of Mathematics and Computer Science), volume 33, 1787–5021

Szegö, G. (1959), Orthogonal polynomials, volume 23 (American Mathematical Soc.)

Williams, P. (2006), A gauss–lobatto quadrature method for solving optimal control problems, *ANZIAM Journal*, 47, 101–115
